# Expanding horizons in burn care: a new paradigm for General Surgeons in Brazil

**DOI:** 10.1590/0100-6991e-20243791-en

**Published:** 2024-08-22

**Authors:** KELLY DANIELLE DE ARAÚJO, ROBERTO MENDES FERREIRA

**Affiliations:** 1 - Fundação Hospitalar do Estado de Minas Gerais, Centro de Tratamento de Queimados Professor Ivo Pitanguy- HJXXIII - Belo Horizonte - MG - Brasil

**Keywords:** Burns, Burn Units, Professional Practice Location, Queimaduras, Unidades de Queimados, Área de Atuação Profissional

## Abstract

The role of the burn surgeon in Burn Treatment Centers (BTCs) is crucial for complementing the multidisciplinary approach in the treatment of burn patients. Globally, the areas of General Surgery and Plastic Surgery are the primary surgical specialties dedicated to this function. The structuring of the Burn Patient Care Line in Minas Gerais highlighted the need to expand the “Burn Care” Field of Expertise, extending it to General Surgery. With the inevitable expansion of the Care Line, pioneered by the state of Minas Gerais, to the federal level, the need for specialized surgical training encompasses both the state context and anticipates the national scenario in the short term. Therefore, the expansion of the “Burn Care” Field of Expertise is fundamental to meeting specific demands and improving the quality of care offered to burn patients, in accordance with international standarts.

## RESUMO

A atuação do cirurgião de queimaduras nos Centros de Tratamento de Queimados (CTQs) é essencial para complementar a abordagem multidisciplinar no tratamento de pacientes queimados. Globalmente, as áreas de Cirurgia Geral e Cirurgia Plástica são as principais especialidades cirúrgicas que se dedicam a essa função. A estruturação da Linha de Cuidado de Assistência ao Paciente Queimado em Minas Gerais destacou a necessidade de ampliar a Área de Atuação “Atendimento ao Queimado” estendendo-a à Cirurgia Geral. Com a inevitável expansão da Linha de Cuidado, pioneiramente estabelecida pelo estado de Minas Gerais, para instância federativa, a necessidade de formação cirúrgica especializada contempla tanto o contexto Estadual como já antevê o cenário Nacional em um futuro de curto prazo. Portanto, a amplificação da Área de Atuação “Atendimento ao Queimado” é fundamental para atender às demandas específicas e melhorar a qualidade do cuidado oferecido aos pacientes queimados, em conformidade com os padrões internacionais.

The speed of social transformations that has been reframing the way of thinking and acting in the contemporary world can be considered one of the consequences of the integration of the internet into citizens’ daily lives. This fact transformed the way of envisioning the mode of Care and Assistance to the health demands that are now guided based on multiprofessional interaction. In the context of care for individuals affected by burns, positive transformations can be observed in the environments of the Burn Treatment Centers (BTCs), evidenced by the articles available on virtual platforms worldwide. The multiprofessional team approach is a successful model in the care of challenging clinical problems such as burns. According to Herndon^1^, since the establishment of the first burn units during and after World War II, advances have been made in the understanding and clinical and surgical treatment of burns, which have reduced post-burn morbidity and mortality, due to actions promoted by horizontally integrated multidisciplinary teams. As discussed by Al-Mousawi, Suman, and Herndon^2^, severe burn management benefits from the concentrated integration of health professionals, with care being amplified by a multiprofessional approach. The complex nature of burn injuries requires a diverse range of skills for optimal care, a single specialist not being expected to possess all the skills and knowledge for such comprehensive approach. Thus, trust is granted to a specialized group to guide integrated care through interdisciplinary organization and collaboration.

In this context emerges the archetype of the Surgeon specialized in burn treatment (Burn Surgeon), who has been contributing to the success of care and assistance in BTCs on several continents. Several authors have published their results asserting Burn Surgeons as effective members of the multiprofessional team of these Centers of Excellence^2^, where the specialty can be exercised by a General or Plastic Surgeon with experience in emergency care and in performing skin debridement and grafts. Their presence is important not only in the initial phase of patient care, since decisions must be made based on physiological responses to injury, current scientific evidence, and appropriate medical/surgical treatments. In addition, this expert must be able to clearly exchange information with a diverse team of experts in other healthcare areas.

Historically, there are some points in the literature to support the objective of this scientific communication. Through a letter to the editor published in the Journal of Burn Care & Rehabilitation, Faucher^3^ addressed the continuing shortage of burn surgeons and its impact on the ability to effectively treat patients with severe burns. According to the author, this was already a persistent concern, with the need of effective professional recruitment and retention strategies. The report of the Conference held in Washington, D.C., in February 2014, coordinated by the former president of the American Burn Association, Dr. Palmer Q. Bessey, maintained focus on the matter, stating the need to improve the training of burn surgeons, justified by the shortage of qualified professionals, the aging of the surgical staff, and the closure of several burn centers due to the lack of specialists^4^. The result indicated that training in burn surgery was often minimized or omitted in general and plastic surgery residencies, which negatively affected residents’ interest in this specialization. Johnson, Jordan, and Shupp^5^ conducted a study in which part of the objectives was to understand the demographics of burn surgeons and job satisfaction in the context of BTCs, predominantly in the United States and Canada. Most participants had a background in General Surgery, followed by postgraduate training in surgical critical care or fellowships in burns. The results indicated that most BTCs train residents of various specialties and that there is a high job satisfaction among burn surgeons, with many recommending the career for young surgeons. However, corroborating the result of the aforementioned Conference, the survey had a response rate of 23%, with only 65 active surgeons and four retired ones participating. Even without consensus, this scenario seems to warrant the need to join multiprofessional efforts to rebalance the state of affairs. This is what happened in 1947 on the “Texas City Disaster”. According to Herndon^1^, from this incendiary catastrophe, considered the largest industrial catastrophe in American history, it was possible to develop the medical team of the “Blocker Burn Unit” in a multidisciplinary way. This is the first burn treatment center in North America to be certified by the American College of Surgeons and the American Burn Association, an accreditation it has maintained continuously since 1996. Leadership or technical responsibility alternates in the medical team between a Plastic Surgeon, a General Surgeon, or a Burn Surgeon, with experience in critical care. The team includes other general and plastic surgeons, intensivists, anesthesiologists, nurses, physical therapists, occupational therapists, nutritionists, and psychosocial specialists (social workers, psychiatrists, and psychologists), among other specialists. The harmony of this “Multiprofessional Orchestra” makes the Blocker Burn Center one that stands out for maintaining the highest survival rate for patients with burns greater than 80% of body surface among all hospitals in the USA1. Moreover, it is recognized for its innovations and research in tissue healing and trauma, as well as for its educational and burn prevention initiatives. 

According to the Association of American Medical Colleges^6^, 26,213 active physicians were registered in general surgery in the United States of America (USA) and there were 7,548 active physicians specializing in plastic surgery in the USA in 2022. Johnson^5^ showed that general surgeons occupy most of the coordination positions in BTCs. In addition, training for Burn Surgery is prevalent among general surgery residents in the USA, which is the fourth largest country and the third most populous in the world. In Brazil, which has the fifth largest territorial extension and is the seventh largest population, Scheffer^8^ registered 41,547 general surgeons and 7,833 plastic surgeons in 2022. When analyzing the number of plastic surgeons in each country, the numbers are very similar. However, in the USA, the workforce in the surgical treatment of burn patients is shared both by general and plastic surgeons, with greater representation of the former.

A survey by the Brazilian Society of Burns in 2021 identified 36 accredited high complexity BTCs in Brazil. Since then, another service has been inaugurated in the state of Maranhão and the state Minas Gerais awaits the activation of five new specialized high complexity services by March 2025, adding to the two already in place and to the nine intermediate complexity ones created in the state. Despite the lack of studies on the shortage of professionals specialized in surgical treatment of burn patients in Brazil, it can be inferred, since Burn Care has been gradually de-accredited due to the lack of filling of the vacancies offered, impacting care and potentially aggravating the situation in the short and medium term. CFM Resolution No. 2,330/2023 - published in the Official Gazette of March 15, 2023, No. 51, Section I, p.112, ratifies CME Ordinance No. 1/2023, which updates the list of specialties and areas of medical practice approved by the Joint Committee on Specialties. In Brazil, currently, 55 medical specialties and 61 areas of expertise are recognized. Among the areas of expertise in medicine, there is the Certification of Area of Expertise in Burn Care, lasting one year, which requires Medical Residency in Plastic Surgery as a prerequisite.

However, the structuring of the multiprofessional team of the Hospital Component, given by Table 02 of Art 19 in Chapter IV of SES/MG Resolution No. 9,075 of October 18, 2023 ^7^, makes clear the need to review the CFM’s resolutive text, dialoguing with international concerns and high-performance services in the treatment of burns outside Brazil. An even more careful view allows us to infer that the structuring of the Line of Care for Burn Patients for the Urgent and Emergency Care Network (ECN) of the State of Minas Gerais seeks to establish a permanent infrastructure that takes advantage of these learnings, while also promoting:

- Protection: Preparing for any sudden increase in demand for burn care, without the need to improvise teams or resources in response to a crisis;
- Ongoing Specialization: Maintaining high standards of care, with the constant presence of specialists and appropriate technology, ensuring that patients receive the best possible treatment in any scenario; and
- Integrated Care: Promotion of collaboration between various specialties and other professionals essential for the treatment of burns.

Consequently, BTCs are encouraged to operate with a readiness and quality mindset that is proactive rather than merely reactive, cementing the multidisciplinary approach as a standard of care. From this perspective, Chapter V of Resolution 9,075, in Table 4 of Article 23, establishes the hierarchy and stratification of risk, determining the correct referral of the patient to the respective BTC hospital size. However, in Brazil it is necessary to implement the changes in a responsible and ethical way. Issues such as the quality and depth of additional training for General Surgeons and the guarantee of specific clinical competence for care of the individual affected by burns are logistical challenges that we must respond to in return for the regulatory standards proposed by the State. In this context, the initiative of the State of Minas Gerais can be seen as an orderly progress in the management of Public Health, if it is accompanied by robust strategies for professional training and certification to ensure the technical and ethical competence of General Surgeons in their new role. In this perspective, Opinion 68.2023 asserts that the Area of Expertise in Burn Care was recently created. Thus, this statement by CRMMG implies that this Area of Activity is still in the consolidation phase, since Burn Care requires a multiprofessional team.

The Burn Care Network in Minas Gerais has been addressed in important events on the national scene, always asserting that Resolution SES/MG No. 9075, of October 18, 2023^7^ is a milestone in the history of burn policies in Brazil, today with annual transfers for co-financing of daily rates in the order of R$ 77 million, and the supply of R$ 23.7 million for the structuring of BTCs with the acquisition of material and equipment. Along the same lines, the expansion of the Area of Expertise in Burn Care was addressed in presentations and panels at the following events by the authors of this scientific communication:

1) XIII Brazilian Congress on Burns, on September 29, 2023, in the city of Salvador, at the pannel “Planning Map for Burn Care”, with the presentation “Care Network”;
2) Reparart-SP, in São Paulo. On October 12, 2023, at the invitation of the Brazilian Society of Plastic Surgery - Regional SP (SBCP-SP), where the theme “Burn Care Network” was also explained;
3) At the 59th Brazilian Congress of Plastic Surgery, held in the city of Campinas on November 18, 2023, the need to expand specialties was pointed out as a prerequisite for the formation of Burn Care, discussed both in the presentation “Burn Care Network” and in the SBCP Burn Chapter Course with the theme “The Situation of Burn Care in Brazil”;
4) At the Plenary of the Regional Council of Medicine of Minas Gerais, on January 4, 2024, the topic “Situation of burn care in Brazil” was discussed, with emphasis on the critical deficiency of specialized professionals in the face of the expansion of the Urgent and Emergency Care Network in the State of Minas Gerais. On this occasion, the CRM-MG declared for taking to the Federal Council of Medicine the discussion of the need to expand the area of activity “Burn Care” to the General Surgeon, and to convene for discussion with the institutions necessary for such an addition; and
5) During the 2024 Minas Brasil Surgery Congress, held on May 2, 2024, in the state capital, Belo Horizonte, the debate on “Burn Care - Training of professionals” was fruitful, involving interinstitutional dialogue with different spheres of leadership and participation of the Public Prosecutor’s Office.

Therefore, the Hospital Foundation of the State of Minas Gerais (FHEMIG) brings the proposal of the pioneering project: Optional Year of General Surgery Residency, optionally extending the time in the formation of the General Surgery Medical Residency Program in the “Burn Care” at BTC Professor Ivo Pitanguy of Hospital João XXIII, in Belo Horizonte. This is the largest Unit in high complexity beds in Brazil. This project was approved by COREME of the HJXXIII on June 6, 2023, and submitted to the MEC inspection on June 19, 2024. As it is a pioneering pilot project in this modality, it cannot yet be called an “Area of Activity”. However, there is a need to expand Certification as an Area of Activity, given the trend of expanding Line of Care that will inevitably reach all the country’s federative states. Thus, the expansion of the Area of Activity is a pioneering initiative by FHEMIG and a proposal for collective interest and Public Health. Therefore, it is essential to convene the various Institutions that together can legitimize the certification of the general surgeon. The objective of this Scientific Communication is to call for the Federal Council of Medicine, the Brazilian Medical Association, the Brazilian College of Surgeons, the Brazilian Society of Plastic Surgery, and the National Commission of Medical Residency, adding to this debate the Brazilian Society of Burns, which can effectively outline the current scenario of burn care in Brazil. We particularly emphasize the need to enable the general surgeon to achieve the certification, to ensure that the standards of care are maintained, that patient safety remains the priority, and that the Line of Care for Burn Patients in Minas Gerais can be implemented. In addition, this initiative will enable the future, duly certified surgeon to work in the BTCs of other Federative States, contributing to specialized care in Brazil, in line with the internationally recognized guidelines and positive experiences.

## References

[1] Herndon DN (2023). The Multidisciplinary Team Approach to Burn Care. Surg Clin North Am.

[2] Al-Mousawi, Ahmed M, Suman, Oscar E, Herndon, David N, HERNDON, David N (2023). Total Burn Care.

[3] Faucher LD (2011). Is There Still a Shortage of Burn Surgeons. J Burn Care Res.

[4] Cochran A, Greenhalgh DG (2018). Building the Burn Physician Workforce for the 21st Century Report From February 2014 Burns Workforce Conference. J Burn Care Res.

[5] Johnson LS, Jordan MH, Shupp JW (2018). Contemplating a Career in Burn Surgery Data From the 2016 Burn Physician Survey. J Burn Care Res.

[6] Association of American Medical Colleges (2023). U.S. Physician Workforce Data Dashboard.

[7] Minas Gerais. Secretaria de Estado de Saúde (2023). Resolução SES/MG n° 9075, de 18 de outubro de 2023. Dispõe sobre a atualização das diretrizes, regras gerais e incentivo de custeio de cofinanciamento da política continuada Linha de Cuidado de Assistência ao Paciente Queimado na Rede de Atenção às Urgências e Emergências do Estado de Minas Gerais, no âmbito da Política de Atenção Hospitalar Valora Minas, e dá outras providências.

[8] Minas Gerais (2022). Secretaria de Estado de Saúde. Deliberação CIB-SUS/MG nº 3.763, de 22 de março de 2022. Aprova as diretrizes de estruturação da Linha de Cuidado da Assistência ao Paciente Queimado para a Rede de Atenção às Urgências e Emergências do Estado de Minas Gerais, no âmbito da Política Hospitalar Valora Minas.

[9] Scheffer M, Guilloux AGA, Miotto BA, Almeida CJ (2023). Demografia Médica no Brasil 2023.

